# Diversity Differences Among Cardiovascular Fellowships Across Five Geographic Regions in the United States

**DOI:** 10.7759/cureus.44217

**Published:** 2023-08-27

**Authors:** Makenzie Dye, Azl Saeed, Trang Q Nguyen, Sarah Yu, Jerome Bey, Dylan Hefner, Casey T Walk, Rebekah Lantz

**Affiliations:** 1 College of Medicine, Wright State University Boonshoft School of Medicine, Fairborn, USA; 2 Surgery, Wright State University, Dayton, USA; 3 Internal Medicine, Miami Valley Hospital, Dayton, USA

**Keywords:** freida, osteopath, gender representation, imgs, us regions, regional disparity, recent data, gme, cardiology fellowship, general cardiology

## Abstract

Introduction

Diversity and inclusion in cardiovascular fellowships are necessary for addressing the healthcare needs of diverse patient populations. However, regional disparities in the diversity of these programs persist, diminishing efforts to create a representative workforce. We observe the regional differences in the diversity of cardiovascular fellowship programs, focusing on gender, doctorate designation, and graduation within the United States (US) or other. We hypothesized that males, medical doctors (MD), and US graduates would be in majority across all regions.

Methods

Data for cardiovascular fellowships from the Fellowship and Residency Electronic Database Access (FREIDA) system for the matriculation year 2022-2023 was obtained to assess the representation of male vs female gender, MD vs osteopathic doctor (DO) designation, and US vs non-US graduate. We then compared these backgrounds to five defined regions (Midwest, Northeast, Southeast, Southwest, and West) in the United States to define representation for backgrounds across geographic areas. Statistical significance was determined by p<0.05 with the use of SAS Studio 3.8, version 9.4 (Cary, NC: SAS Institute, Inc.), and Wilson score for confidence intervals.

Results

We found significant disparities across all background factors for all regions. This includes that females, DOs, and non-US graduates were underrepresented among Midwest, Northeast, Southeast, Southwest, and West regions, and the p-value was <0.001 for all variations. Specifically for Midwest, the female frequency was 155 (23.81%; CI: 21, 27; p<0.001), DO frequency was 101 (15.51%; CI: 13, 19; p<0.001), and non-US graduate frequency was 206 (31.84%; CI: 28, 36; p<0.001). For Northeast, the female frequency was 231 (29.62; CI: 27, 33; p<0.001), DO frequency was 72 (9.22; CI: 7, 11; p<0.001), and non-US graduate frequency was 239 (30.68; CI 28, 34; p<0.001). For Southeast, the female frequency was 178 (25.99; CI: 23, 29; p<0.001), DO frequency was 67 (9.78; CI: 8, 12; p<0.001), and non-US graduate frequency 279 (41.46; CI: 38, 45; p<0.001). For Southwest, the female frequency was 74 (26.71; CI: 22, 32; p<0.001), DO frequency was 21 (7.58; CI 5, 11; p<0.001), and non-US graduate frequency was 110 (39.71; CI: 34,46; p<0.001). For West, the female frequency was 107 (31.75; CI 27, 37; p<0.001), DO frequency was 15 (4.45; CI: 3, 7; p<0.001), and non-US graduate frequency was 54 (16.07; CI: 13, 20; p<0.001).

Conclusion

We emphasize the regional disparities for females, DOs, and non-US graduates within cardiovascular fellowships in the past matriculation year. Understanding that we have not reached diversity goals allows for further reflection and implementation of targeted interventions and initiatives aimed at promoting equal opportunities for applicants. This is true for all regions of the United States. By addressing these disparities, fellowship programs can more effectively mirror the diverse patient populations they serve and foster a healthcare environment that is inclusive and accommodating. This, in turn, contributes to the overall enhancement of healthcare outcomes.

## Introduction

The overall United States (US) patient population has continued to diversify in recent years. Admissions to medical schools have mirrored this pattern; nevertheless, as training levels increase, the inclusion of various demographic groups shows a progressively evident decrease leading to a situation where males, medical doctors (MD), and graduates from the US hold a competitive edge over females, doctors of osteopathic medicine (DO), and their international medical graduate counterparts. This is especially true for cardiovascular fellowships.

Slightly over half of the US medical students are female, which is consistent with population trends, meanwhile, only 21% are cardiovascular fellows [1,2]. Progressively, 7% of interventional cardiology and 6% of electrophysiology fellows are female [2]. This is true for other career advancements, where women are less likely to be medical faculty, full professors, hospital chief executive officers (CEOs), and deans and department chairs [3,4]. Richter et al. follow a sample of 559,098 medical students from 134 medical schools through their careers from 1979 to 2013 and faculty data through 2018. They noted that female counterparts were less likely than their male peers to achieve the following designations: associate professor (hazard ratio {HR}: 0.76) (confidence interval {CI}: 0.74, 0.78), full professor (HR: 0.77; CI: 0.74, 0.81), or department chair (HR: 0.46; CI: 0.39, 0.54). Interestingly, sex differences in promotions and appointments did not improve toward gender representation over time. There were no differences for the later sample of students than for the earlier sample of students through their medical careers [3].

Similarly, Ohio State University performed a study in 2021 for a collection period of April-May that year to reveal more information about gender roles and predict the factors associated with having a female CEO. Executive positions at five affiliated hospitals, health insurance groups, and the US Department of Health and Human Services were obtained in their study. Of 3911 senior executives from health systems and health insurance groups, 3462 boards of directors (BODs) within these groups, and 31 leadership positions within the US Department of Health and Human Services were obtained to reveal female representation was 17.5% on BOD and 15.3% CEO for health systems and 21.3% on BOD and 15.8% CEO for health insurance groups. The US Department of Health and Human Services did slightly better with women (N=18, 18/31=58.1%). The conclusion of the study notes that despite representing a slight majority of the US population, women remain underrepresented on leadership teams, with implications on policy decisions toward female health. When females represent BOD and executive positions, they may be more likely to pick a female candidate to fulfill further roles [4]. Despite women starting with good representation in early careers at this time, as they progress, it is now well understood that they face a disadvantage in further male-dominant specialties, such as cardiology-related fields, and if they have ambitious career and leadership goals.

DOs are trained similarly to MDs in clinical medicine, with the addition of a holistic aspect of healthcare that addresses the patient as a mind, body, and soul beyond the persistent addition of medications to treat chronic illnesses. Trainees of the discipline also learn 200 hours of non-invasive body alignment, vascular, and lymphatic techniques to enhance their approach to patient care, which some patients may prefer [5,6]. However, for matching to advanced training programs, matriculation rates appear to favor MDs over DOs [6]. DOs often need to sit and pay for twice the amount of certification exams to emphasize their value to advanced training programs, at the expense of additional time and finances compared to MD peers [7].

Dye and Lantz previously discuss some of the difficulties that non-US patients and medical graduates encounter when navigating a new healthcare system including visa permissions and interpersonal trust [8]. Mannerisms, faith, and cultural normalcies are important in this composition of change and perceptions of the healthcare system. Additionally, those pursuing a medical career fall into the niche of primary care specialty, in part by program acceptance and perhaps by the desire to treat patients in underserved regions [8]. However, it should be noted that they come to the United States with these several challenges to match into competitive programs, such as cardiology.

Given the current knowledge of disparity, we wished to observe the latest information provided by the Fellowship and Residency Electronic Database Access (FREIDA) system regarding the matriculation year 2022-2023 for readily identifiable backgrounds of fellows by using their accredited programs as our guide [9]. We previously published our analysis regarding cardiovascular disparities [10], and we wished to further investigate these trends by the five standard US geographic regions [11]. Additionally, our data may inadvertently assist diverse applicants with their most likely to match regions based on personal background details.

This study will be presented by the third author as an abstract at CHEST 2023 in Hawaii, taking place in October 2023.

## Materials and methods

Data for cardiovascular fellowships from FREIDA, the American Medical Association’s (AMA) residency and fellowship database, were obtained for the matriculation year 2022-2023 to assess the current distribution of three background details of fellows [9]. Our goal was to identify a statistical significance from 50% representation for male/female gender, MD/DO training, and US/non-US graduate status. Fellows in accredited Accreditation Council for Graduate Medical Education (ACGME) programs were included. If demographics were unknown due to lack of information on the fellowship page or if the program was unaccredited or newly accredited, individuals and programs were respectively excluded in order to maintain our statistical integrity.

The first and second authors collected initial data for cardiovascular programs. We also assessed other cardiology-related background fellowships, such as interventional cardiology, electrophysiology, and heart failure and transplant, to reflect internal medicine-derived disciplines. Additionally, we observed data for vascular and thoracic surgery (including cardiothoracic surgery). Data on regions were only significant for the large cardiovascular population given the other internal medicine disciplines derived from this preliminary fellowship. We did not obtain data for general surgery or vascular and thoracic-integrated programs, therefore there was not sufficient data for these surgical backgrounds.

We defined our data to the five recognized geographic US regions: Midwest, Northeast, Southeast, Southwest, and West [11]. Midwestern states include Iowa (IA), Illinois (IL), Indiana (IN), Kansas (KS), Michigan (MI), Minnesota (MN), Missouri (MO), Nebraska (NE), North Dakota (ND), Ohio (OH), South Dakota (SD), and Wisconsin (WI). There were no accredited programs for ND. Northeastern states include Connecticut (CT), Massachusetts (MA), Maine (ME), Pennsylvania (PA), New Hampshire (NH), New Jersey (NJ), New York (NY), Rhode Island (RI), and Vermont (VT). Southeastern states include Alabama (AL), Arkansas (AR), Delaware (DE), Florida (FL), Georgia (GA), Kentucky (KY), Louisiana (LA), Maryland (MD), Mississippi (MS), North Carolina (NC), South Carolina (SC), Tennessee (TN), Virginia (VA), and West Virginia (WV). We also included the District of Columbia (DC) in this analysis. Southwestern states include Arizona (AZ), Oklahoma (OK), New Mexico (NM), and Texas (TX). Western states include Alaska (AK), California (CA), Colorado (CO), Hawaii (HI), Idaho (ID), Montana (MT), Nevada (NV), Oregon (OR), Utah (UT), Washington (WA), and Wyoming (WY). There were no accredited programs in AK, ID, MT, or WY.

For each variable, male/female, DO/MD, and US/non-US graduate, a binomial test of proportions was used to determine whether any of the percentages were significantly different (p<0.05). Statistical analyses were performed using SAS Studio 3.8, version 9.4 (Cary, NC: SAS Institute, Inc.) and confidence intervals were calculated via the Wilson score interval procedure [12]. We hypothesized that females, DOs, and non-US graduates would have underrepresentation compared to male, MD, and US graduate peers and that this may occur for all US regions. We suspected that the Midwest may represent females and DOs equally, given the amount of perceived medical training facilities and training opportunities. We hypothesized that the coastal states and non-continental states, Hawaii (there were no accredited programs in Alaska), would reflect representation for non-US graduates due to increased geographical exposure to Asia. Our results were interpreted as groups by region and further separated into individual states for closer analysis.

## Results

In our analysis, we identified 243 programs listed in FRIEDA, each associated with a program director. We were able to include 186 (77%) of these, accounting for three demographics of our study. Fifty-six (23%) programs were unable to be included due to lacking one or more gender, MD/DO designation, or US graduate status. Females, DOs, and non-US graduates were statistically underrepresented in all US geographic regions (Table 1). We further observed trends in specific states and did note some trends as delineated in Table 2.

**Table 1:** Diversity in general cardiology by US region data. DO: doctor of osteopathy

Region	Variable	Frequency (%)	95% CI (%)	p-value
Midwest	Female gender	155 (23.81)	21, 27	<0.0001
	Male gender	496 (76.19)		
	DO specialty	101 (15.51)	13, 19	<0.0001
	MD specialty	550 (84.49)		
	Non-US graduate	206 (31.84)	28, 36	<0.0001
	US graduate	441 (68.16)		
Northeast	Female gender	231 (29.62)	27, 33	<0.0001
	Male gender	549 (70.38)		
	DO specialty	72 (9.22)	7, 11	<0.0001
	MD specialty	709 (90.78)		
	Non-US graduate	239 (30.68)	28, 34	<0.0001
	US graduate	540 (69.32)		
Southeast	Female gender	178 (25.99)	23, 29	<0.0001
	Male gender	507 (74.01)		
	DO specialty	67 (9.78)	8, 12	<0.0001
	MD specialty	618 (90.22)		
	Non-US graduate	279 (41.46)	38, 45	<0.0001
	US graduate	394 (58.54)		
Southwest	Female gender	74 (26.71)	22, 32	<0.0001
	Male gender	203 (73.29)		
	DO specialty	21 (7.58)	5, 11	<0.0001
	MD specialty	256 (92.42)		
	Non-US graduate	110 (39.71)	34, 46	<0.0001
	US graduate	167 (60.29)		
West	Female gender	107 (31.75)	27, 37	<0.0001
	Male gender	230 (68.25)		
	DO specialty	15 (4.45)	3, 7	<0.0001
	MD specialty	322 (95.55)		
	Non-US graduate	54 (16.07)	13, 20	<0.0001
	US graduate	282 (83.93)		

**Table 2:** Further detailed breakdown of state diversity in general cardiology by region. AL: Alabama; AZ: Arizona; AR: Arkansas; CA: California; CO: Colorado; CT: Connecticut; DC: District of Columbia (nation’s capital); DE: Delaware; FL: Florida; GA: Georgia; HI: Hawaii; IL: Illinois; IA: Iowa; KS: Kansas; KY: Kentucky; LA: Louisiana; ME: Maine; MD: Maryland; MI: Michigan; MN: Minnesota; MS: Mississippi; MO: Missouri; NE: Nebraska; NV: Nevada; NH: New Hampshire; NJ: New Jersey; NM: New Mexico; NY: New York; NC: North Carolina; OH: Ohio; OK: Oklahoma; OR: Oregon; PA: Pennsylvania; RI: Rhode Island; SC: South Carolina; SD: South Dakota; TN: Tennessee; TX: Texas; UT: Utah; VT: Vermont; VA: Virginia; WA: Washington; WI: Wisconsin States not represented here are Alaska, Idaho, Montana, North Dakota, and Wyoming.

US region		Female frequency (%)	DO frequency (%)	Non-US graduate frequency (%)
Midwest	IA	6 (17.14)	4 (11.43)	18 (51.43)
	IL	28 (28)	13 (13)	30 (30.61)
	IN	3 (14.29)	3 (14.29)	1 (4.76)
	KS	5 (26.32)	3 (15.79)	12 (63.16)
	MI	43 (29.45)	45 (30.82)	40 (27.40)
	MN	11 (17.19)	1 (1.56)	17 (26.56)
	MO	11 (21.57)	1 (1.96)	15 (29.41)
	NE	8 (38.10)	5 (23.81)	14 (66.67)
	OH	29 (21.17)	13 (9.49)	40 (29.20)
	SD	0	0	7 (100)
	WI	11 (22)	13 (26)	12 (24)
Northeast	CT	24 (42.86)	5 (8.77)	33 (60)
	MA	43 (35.83)	8 (6.67)	44 (36.67)
	ME	1 (11.11)	1 (11.11)	1 (11.11)
	PA	69 (31.94)	34 (15.74)	66 (30.56)
	NH	4 (18.57)	0	1 (7.14)
	NJ	13 (18.84)	15 (21.74)	8 (11.59)
	NY	65 (24.44)	8 (3.01)	82 (30.83)
	RI	8 (44.44)	0	2 (11.11)
	VT	4 (33.33)	1 (8.33)	2 (16.67)
Southeast	AL	6 (20.69)	0	10 (34.48)
	AR	4 (30.77)	0	9 (69.23)
	DC	7 (29.17)	0	7 (29.17)
	DE	2 (20)	3 (30)	5 (50)
	FL	46 (32.17)	18 (12.59)	61 (43.89)
	GA	8 (28.57)	5 (17.86)	16 (57.14)
	KY	8 (18.60)	13 (30.23)	20 (46.51)
	LA	18 (27.27)	2 (3.03)	30 (45.45)
	MD	13 (28.89)	0	14 (31.11)
	MS	3 (14.29)	4 (19.05)	6 (28.57)
	NC	22 (27.85)	22 (27.85)	20 (25.32)
	SC	7 (28)	7 (28)	5 (20)
	TN	18 (27.69)	18 (27.69)	25 (38.46)
	VA	12 (20.34)	12 (20.34)	22 (37.29)
	WV	4 (11.43)	4 (11.43)	31 (88.57)
Southwest	AZ	11 (33.33)	2 (6.06)	14 (42.42)
	OK	2 (11.76)	8 (47.06)	5 (29.41)
	NM	6 (46.15)	1 (7.69)	2 (15.38)
	TX	55 (25.70)	10 (4.67)	89 (41.59)
West	CA	80 (35.24)	4 (1.76)	26 (11.50)
	CO	9 (32.14)	5 (17.86)	6 (21.43)
	HI	2 (22.22)	1 (11.11)	2 (22.22)
	NV	1 (10)	0	8 (80)
	OR	4 (21.05)	4 (21.05)	6 (31.58)
	UT	6 (30)	1 (5)	4 (20)
	WA	5 (20.83)	0	2 (8.33)

Midwest

In the Midwest, the female frequency was 155 (23.81; CI: 21, 27; p<0.001), DO frequency was 101 (15.51; CI: 13, 19; p<0.001), and non-US graduate frequency was 206 (31.84; CI: 28, 36; p<0.001). All backgrounds were underrepresented.

By-state data showed that Iowa female frequency was 6 (17.14), DO frequency was 4 (11.43), non-US graduate frequency was 18 (51.43); Illinois female frequency was 28 (28), DO frequency was 13 (13), non-US graduate frequency was 30 (20.61); Indiana female frequency was 3 (14.29), DO frequency was 3 (14.29), non-US graduate frequency was 1 (4.76); Kansas female frequency was 5 (26.32), DO frequency was 3 (15.79), non-US graduate frequency was 12 (63.16); Michigan female frequency was 43 (29.45), DO frequency was 45 (30.82), non-US graduate frequency was 40 (27.40); Minnesota female frequency was 11 (17.19), DO frequency was 1 (1.59), non-US graduate frequency was 17 (26.56); Missouri female frequency was 11 (21.57), DO frequency was 1 (1.96), non-US graduate frequency was 15 (29.41); Nebraska female frequency was 8 (38.10), DO frequency was 5 (23.81), non-US graduate frequency was 14 (66.67); Ohio female frequency was 29 (21.17), DO frequency was 13 (9.49), non-US graduate frequency was 40 (29.20); South Dakota female frequency was 0, DO frequency was 0, non-US graduate frequency was 7 (100); Wisconsin female frequency was 11 (22), DO frequency was 13 (26), non-US graduate frequency was 12 (24).

Among the Midwest states, Nebraska had the highest percentage of female fellows (38.10%), followed by Michigan (29.45%) and Illinois (28%). South Dakota had the lowest percentage of female cardiology fellows in the Midwest region of the United States with no female fellows. Michigan had the highest percentage of DO fellows (30.82%), followed by Nebraska (23.81%) and Wisconsin (26%). South Dakota was completely represented by non-US graduates. Other states with high non-US graduate percentages included Nebraska (66.67%) and Kansas (63.16%).

Northeast

In the Northeast, female frequency was 231 (29.62; CI: 27, 33; p<0.001), DO frequency was 72 (9.22; CI: 7, 11; p<0.001), non-US graduate frequency was 239 (30.68; CI: 28, 34; p<0.001). All backgrounds were underrepresented.

By-state data showed that Connecticut female frequency was 24 (42.86), DO frequency was 5 (8.77), non-US graduate frequency was 33 (60); Massachusetts female frequency was 43 (35.83), DO frequency was 8 (6.67), non-US graduate frequency was 44 (36.67); Maine female frequency was 1 (11.11), DO frequency was 1 (11.11), non-US graduate frequency was 1 (11.11); Pennsylvania female frequency was 69 (31.94), DO frequency was 34 (15.74), non-US graduate frequency was 66 (30.56); New Hampshire female frequency was 4 (18.57), DO frequency was 0, non-US graduate frequency was 1 (7.14); New Jersey female frequency was 13 (18.84), DO frequency was 15 (21.74), non-US graduate frequency was 8 (11.59); New York female frequency was 65 (24.44), DO frequency was 8 (3.01), non-US graduate frequency was 82 (30.83); Rhode Island female frequency was 8 (44.44), DO frequency was 0, non-US graduate frequency was 2 (11.11); Vermont female frequency was 4 (33.33), DO frequency was 1 (8.33), non-US graduate frequency was 2 (16.67).

Among the Northeast states, Rhode Island had the highest frequency of female fellows (44.44%), followed by Connecticut (42.86%), and Massachusetts (35.83%). Maine had the least percentage of female fellows (11.11%). New Jersey had the highest percentage of DO fellows (21.74%), followed by Pennsylvania (15.74%) and Maine (11.11%). New Hampshire and Rhode Island demonstrated no representation for DOs. Connecticut had the highest percentage of non-US graduates (60%), followed by Maine (36.67%) and New York (30.83%).

Southeast

In the Southeast, female frequency was 178 (25.99; CI: 23, 29; p<0.001), DO frequency was 67 (9.78; CI: 8, 12; p<0.001), and non-US graduate frequency was 279 (41.46; CI: 38, 45; p<0.001). All backgrounds were underrepresented.

By-state data showed that Alabama female frequency was 6 (20.69), DO frequency was 0, non-US graduate frequency was 10 (34.48); Arkansas female frequency was 4 (30.77), DO frequency was 0, non-US graduate frequency was 9 (69.23); District of Columbia female frequency was 7 (29.17), DO frequency was 0, non-US graduate frequency was 7 (29.17); Delaware female frequency was 2 (20), DO frequency was 3 (30), non-US graduate frequency was 5 (50); Florida female frequency was 46 (32.17), DO frequency was 18 (32.17), non-US graduate frequency was 61 (43.89); Georgia female frequency was 8 (28.57), DO frequency was 5 (17.86), non-US graduate frequency was 16 (57.14); Kentucky female frequency was 8 (18.60), DO frequency was 13 (30.23), non-US graduate frequency was 20 (46.51); Louisiana female frequency was 18 (27.27), DO frequency was 2 (3.03), non-US graduate frequency was 30 (45.45); Maryland female frequency was 13 (28.89), DO frequency was 0, non-US graduate frequency was 14 (31.11); Mississippi female frequency was 3 (14.29), DO frequency was 4 (19.05), non-US graduate frequency was 6 (28.57); North Carolina female frequency was 22 (27.85), DO frequency was 2 (2.53), non-US graduate frequency was 20 (25.32); South Carolina female frequency was 7 (28), DO frequency was 4 (16), non-US graduate frequency was 5 (20); Tennessee female frequency was 18 (27.69), DO frequency was 10 (15.38), non-US graduate frequency was 25 (38.46); Virginia female frequency was 12 (20.34), DO frequency was 4 (6.78), non-US graduate frequency was 22 (37.29); West Virginia female frequency was 4 (11.43), DO frequency was 2 (5.71), non-US graduate frequency was 31 (88.57).

Among the Southeast states, Florida had the highest percentage of female fellows (32.17%), followed by Arkansas (30.77%) and the District of Columbia (29.17%). West Virginia had the least percentage of female fellows (11.43%). Kentucky had the highest percentage of DO fellows (30.23%), followed by Delaware (30%) and Mississippi (19.05%). There was no DO representation in Alabama, Arkansas, the District of Columbia, or Maryland. West Virginia had the highest percentage of non-US graduates (88.59%), followed by Arkansas (69.23%) and Georgia (57.14%).

Southwest

In the Southwest, female frequency was 74 (26.71; CI: 22, 32; p<0.001), DO frequency was 21 (7.58; CI 5, 11; p<0.001), non-US graduate frequency was 110 (39.71; CI: 34, 46; p<0.001). All backgrounds were underrepresented.

By-state data showed that Arizona female frequency was 11 (33.33), DO frequency was 2 (6.06), non-US graduate frequency was 14 (42.42); Oklahoma female frequency was 2 (11.76), DO frequency was 8 (47.06), non-US graduate frequency was 5 (29.41); New Mexico female frequency was 6 (46.15), DO frequency was 1 (7.69), non-US graduate frequency was 2 (15.38); Texas female frequency was 55 (25.70), DO frequency was 10 (4.67), non-US graduate frequency was 89 (41.59).

Among the Southwest states, New Mexico had the highest frequency of female fellows (46.15%), followed by Arizona (33.33%), and Texas (25.70%). Oklahoma had the least percentage of female fellows (11.76%). Oklahoma had the highest percentage of DO fellows (47.06%), followed by New Mexico (7.69%) and Arizona (6.06%). Texas had the least percentage of DOs (4.67%). Arizona had the highest percentage of non-US graduates (42.42%), followed by Texas (41.59%) and Oklahoma (29.41%). New Mexico had the least percentage of non-US graduates (15.38%).

West

In the West, female frequency was 107 (31.75; CI: 27, 37; p<0.001), DO frequency was 15 (4.45; CI: 3, 7; p<0.001), non-US graduate frequency was 54 (16.07; CI: 13, 20; p<0.001). All backgrounds were underrepresented.

By-state data showed that California female frequency was 80 (35.24), DO frequency was 4 (1.76), non-US graduate frequency was 26 (21.43); Colorado female frequency was 9 (32.14), DO frequency was 5 (17.86), non-US graduate frequency was 6 (21.43); Hawaii female frequency was 2 (22.22), DO frequency was 1 (11.11), non-US graduate frequency was 2 (22.22); Nevada female frequency was 1 (10), DO frequency was 0, non-US graduate frequency was 8 (80); Oregon female frequency was 4 (21.05), DO frequency was 4 (21.05), non-US graduate frequency was 6 (31.58); Utah female frequency was 6 (30), DO frequency was 1 (5), non-US graduate frequency was 4 (20); Washington female frequency was 5 (20.83), DO frequency was 0, non-US graduate frequency was 2 (8.33).

**Figure 1:**
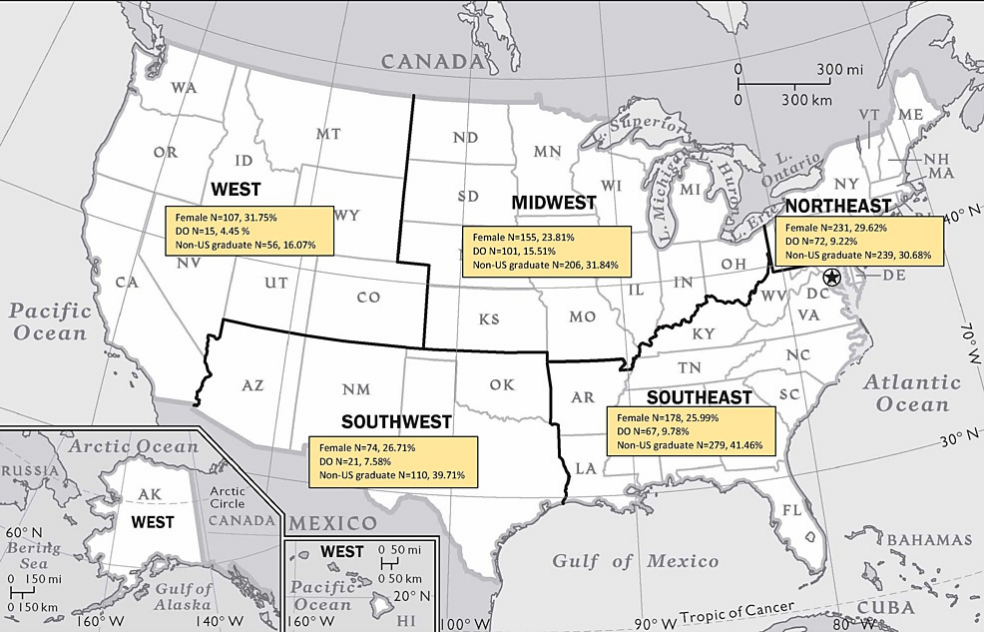
Distribution of diversity across US geographic regions (adopted per peer-review recommendations). The image is adapted with permission from the National Geographic Society [11].

Among the West states, California had the highest frequency of female fellows (35.24%), followed by Colorado (32.14%), and Utah (30%). Nevada had the least percentage of female fellows (10%). Oregon had the highest percentage of DO fellows (21.05%), followed by Colorado (17.86%) and Hawaii (22.22%). Nevada and Washington had no DO fellows. Nevada had the highest percentage of non-US graduates (80%), followed by Oregon (31.58%) and Hawaii (22.22%). Washington had the least percentage of non-US graduates (8.33%). Respective distributions for regions are shown in the geography map in Figure 1.

## Discussion

Understanding the current landscape within cardiology is essential for developing effective strategies to promote diversity and inclusion in the future. It is crucial to address disparities in the distribution of cardiovascular fellowships to ensure that patients from varying geographic locations receive equal access to high-quality care. We collected data on cardiovascular fellow diversity across geographic regions of the United States and found that all regions demonstrated disparities for females, DOs, and non-US graduates with a significance of p<0.0001 for all variables. However, current data show that applicants may have been more likely to match for 2022-2023 in some of the following distributions and we suspect this may be a trend, which would be interesting to observe in future studies. (1) If they are female in the West, the frequency was 107 (31.75%; CI 27, 37), especially in California (35.24%). (2) If they are DO in the Midwest, the frequency was 101 (15.51; CI 13, 19), especially in Michigan (30.82%). (3) If they are non-US graduates in the Southeast, the frequency was 279 (41.46; CI 38, 45), especially in West Virginia (88.59%).

Our analysis is consistent with previous literature that also shows disparities in programs, regions, and state-level perceptions from cardiovascular program directors (PDs). Crowley et al. in the Journal of the American Heart Association, noted that PDs lack a perceived necessity to increase diversity within their program. Sixty-three percent of cardiovascular PDs do not think that diversity needs to be increased in their program, and only 6% ranked diversity or the ability to enhance cultural competency as a “top 3” priority when making their fellowship rank list [12,13]. Moreover, even among the 37% of PDs who wish to increase diversity within their home program, <50% have a strategy in place to accomplish this objective [13].

Unfortunately, resistance to increased diversity in cardiovascular training can have a significant negative impact on patient outcomes. Diversity among healthcare providers is associated with better patient outcomes and reduced health disparities. For example, female physicians tend to engage in more patient-centered communication and provide health counseling more often than their male counterparts [14]. Additionally, patients who receive care from female providers are more likely to receive treatment for heart failure that aligns with established guidelines [15]. Other research has also shown the positive benefits of increased diversity of underrepresented minorities, who are more likely to care for underserved populations than physicians who belong to majority groups [15]. Additionally, other studies have shown race-dependent concordance and increased likelihood of consent to both preventive health services and heart surgery if the recommending physician is also underrepresented in medicine [16,17].

Diverse training environments improve important skills for physicians, such as thinking, empathy, and motivation [18], which improves patient health equity and downstream patient outcomes [19,20]. Unfortunately, despite the advantages in patient and provider experiences, only a third of cardiovascular PDs have current plans to actively increase diversity in their programs. There are other barriers believed to be outside the control of an admission committee that hinder attempts to diversify groups of trainees [8,20]. If trainees do not see people like themselves represented in programs, they may be less likely to select programs, knowing that they may be faced with more challenges compared to their peers [8]. The suggestion from Bhasin et al. involves the cooperation of undergraduate and post-graduate administrators to create a "deep pipeline" through universities, high schools, and elementary schools to enhance and recruit interest among diverse minds and backgrounds in the field [16]. The idea would be to garner numbers of interested persons, as a stepping block for fellowships to diversify. Among other opportunities for improvement are inclusion in academic medicine and leadership roles [14].

Cardiovascular trainees eventually progress to attending physicians, and without a diverse pool of providers who can understand and respect differences that are important to patients, patients may not receive the personalized care they need [20]. By increasing diversity in cardiovascular programs, healthcare organizations can help to ensure that patients receive high-quality care that improves outcomes and reduces health disparities.

Strengths

Strengths of our study include that it collects the most recent FREIDA data, which allows for up-to-date analysis and comparison to prior fellow-in-training results. We were able to include 77% of the fellowship information. To our knowledge, no previous study has assessed fellow data for cardiovascular fellowship by US geographic region for gender, MD/DO status, and US/non-US graduate comparisons.

Limitations

The limitations of our study encompass the fact that although examining trends over a shorter duration can yield valuable insights, scrutinizing data over an extended period can furnish a more holistic comprehension of patterns and fluctuations. In the future, it may be of benefit to incorporate more extended periods of data analysis to gain a deeper understanding of trend evolution over time. This study only contains data for trainees; however, it may be beneficial to observe the diversity of practicing attendings. Additionally, this study excluded recently accredited programs because there was a lack of fellow data available which would skew the data but may be of use to include in future cross-sectional and longitudinal studies.

In conclusion, disparities in diversity persist for females, DOs, and non-US graduates in cardiovascular fellowship. This is consistent with prior studies where trends, notably in gender representation, have not improved. We take this a step further and observe the latest data on DO and non-US graduate representation compared to MD and US graduate peers and note the same, significant disparities in representation by US geographic regions. There are steps programs and PDs can take to improve this important attribute to patient health outcomes by collaborating with pre-graduate medical education (GME) at the elementary, high school, and undergraduate levels, in order to garner interest in cardiovascular medicine in underrepresented areas of the country. Selection committees can include diverse panels in their admission committee and incorporate multiple views into their decisions for residents and fellow candidates. Research and leadership opportunities can be offered at earlier stages, with the inclusion of underrepresented persons. This same mentality may be continued into later career board-of-director and executive positions, again with the purpose of improving population health and patient health outcomes.

## Conclusions

We wished to assess diversity in cardiovascular fellowships in the latest matriculation year and provide regional analysis for gender, medical school degree, and US graduate status. We show the underrepresentation of female, DO, and non-US graduate trainees that persist within cardiology, ultimately emphasizing the importance of increased diversity among fellows across US regions at a national level. By addressing disparities through targeted interventions and support for underrepresented groups, we can improve the quality of care for patients and work towards a more equitable healthcare system. It is essential to prioritize diversity and inclusion in medicine and strive for a future in which historically underrepresented groups will have greater representation.
